# The Application of Lipid Membranes in Biosensing

**DOI:** 10.3390/membranes8040108

**Published:** 2018-11-14

**Authors:** Georgia-Paraskevi Nikoleli, Dimitrios P. Nikolelis, Christina G. Siontorou, Marianna-Thalia Nikolelis, Stephanos Karapetis

**Affiliations:** 1 Laboratory of Inorganic & Analytical Chemistry, School of Chemical Engineering, Dept 1, Chemical Sciences, National Technical University of Athens, 9 Iroon Polytechniou St., 15780 Athens, Greece; stevekara@chem.uoa.gr; 2 Laboratory of Environmental Chemistry, Department of Chemistry, University of Athens, Panepistimiopolis-Kouponia, 15771 Athens, Greece; nikolelis@chem.uoa.gr (D.P.N.); m.nikolelis@gmail.com (M.-T.N.); 3 Laboratory of Simulation of Industrial Processes, Department of Industrial Management and Technology, School of Maritime and Industry, University of Piraeus, 18534 Pireus, Greece; csiontor@unipi.gr

**Keywords:** biosensors, lipid membrane-based devices, food analysis, environmental monitoring, nanotechnology

## Abstract

The exploitation of lipid membranes in biosensors has provided the ability to reconstitute a considerable part of their functionality to detect trace of food toxicants and environmental pollutants. This paper reviews recent progress in biosensor technologies based on lipid membranes suitable for food quality monitoring and environmental applications. Numerous biosensing applications based on lipid membrane biosensors are presented, putting emphasis on novel systems, new sensing techniques, and nanotechnology-based transduction schemes. The range of analytes that can be currently using these lipid film devices that can be detected include, insecticides, pesticides, herbicides, metals, toxins, antibiotics, microorganisms, hormones, dioxins, etc. Technology limitations and future prospects are discussed, focused on the evaluation/validation and eventually commercialization of the proposed lipid membrane-based biosensors.

## 1. Introduction

Biosensors, in general, translate a chemical or biochemical interaction into a signal, e.g., voltage, current, absorbance, etc. The sector is very dynamic, continuously evolving and well established, almost in all continents, with remarkable infrastructure and human potential. Biosensors have a large number of applications in food analysis and environmental monitoring and provide distinct advantages as compared to liquid and gas chromatographic techniques such as fast response times, portability, high sensitivity and selectivity, very small preparation of sample, etc. There is a clear difference between the multiple-use and single-shot because the latter characterizes the devices that are used only for one test. Nanosensing currently involves many research areas, of which the most important are the field of nano-material-based biosensors.

Since Mueller and colleagues’ pioneered work on bilayer lipid membranes (BLMs) [1], the number of biosensor devices based on lipid films for applications in food toxicants detection or environmental pollutants monitoring has tremendously increased. However, the so called “black” lipid films produced were very fragile and were prone to electrical and mechanical breakage and were not stable outside an electrolyte solution. This has prohibited their practical applications. Lipid membranes-based biosensors represent an appropriate biocompatible structure with rapid response times, high sensitivity and selectivity, small size, and portability, and offer many advantages compared with the bulky analytical instrumentation such as liquid chromatographic units. The new generation of stabilized lipid membrane nanosensors has the potential to develop site-specific monitors with respect to analytical performance, operational stability, and response. 

Recent review articles that explore and report various platforms of lipid membranes and their applications in biosensing have been given in the literature by Janshoff and Steinem [2]. The first attempt to construct bilayer lipid membranes (BLMs) was made 30 years ago [3]. However, these membranes (the so called “black lipid films”) retained the solvent in their structure and the results were not reproducible. Methods for the preparation of solvent-free lipid membranes have also appeared in the literature [4], but their major drawback was that these films were highly unstable and were prone to collapse by a mechanical or electrical shock. This major drawback of these lipid films was no longer a problem because methods to improve the stability were reported in the literature such as interconnected membrane bubbles in organic solvents without a substrate aperture [5,6], or through the so called “monolayer technique”.

Lipid membrane-based devices have been explored for the last three decades in biosensors. However, their stability has prohibited their practical applications. Recent developments in the development of stabilized lipid membrane devices have offered the opportunity for practical implementation of this class of biosensors. More specifically, incorporation of stabilized lipid films on graphene and ZnO electrodes has offered a range of strategies, architectures, and materials for biomedical sensing, for example increasing drastically to include non-enzymatic catalysis schemes and other prototype schemes of detection. Therefore, the number of affordable devices has increased tremendously and have been integrated into systems for market applications. These applications include a large number of food toxicants and environmental pollutants, such as cholera toxin, aflatoxin M1 and B1, saxitoxin, carbamates, arochlor 1242, hydrazines, naphthalene acetic acid (NAA), doping materials (such as dopamine, adrenaline and ephedrine), urea, uric acid, etc. 

This work reviews the devices based on lipid films that were explored for applications in various fields of science such as for biomedical applications, food analysis, and environmental monitoring. The paper provides novel reports on the design and microfabrication of prototype lipid membrane nanosensing devices for the rapid field detection of food toxicants and environmental pollutants, for biomedical applications and the challenges that lie ahead. Reviews on this important issue of science have been published [7], however, in the present article we provide novel achievements on graphene and ZnO electrodes-based nanosensors that use filter-supported lipid membranes and their utilization to offer devices for commercialization. 

## 2. The Preparation of Lipid Membranes

The methods of the preparation of lipid membranes are summarized as follows:

- Black lipid films
- Solvent less lipid films
  Both the above lipid membranes belong to the category of free-standing BLMs.
- Supported lipid membranes on (i) metal (ii) silicon (iii) glass fiber
- Polymerized supported lipid membranes
  Both (c) and (d) are classified to the supported lipid membranes.

## 3. Methods for Preparation Biosensors Based on Lipid Films

Over the last two decades, a variety of techniques have been proposed for the construction of stabilized lipid membranes that are not susceptible to electrical or mechanical failure [8,9,10,11]. Most of these techniques provide lipid membranes that are stable enough for practical applications, whereas their less than 1 µm size can describe the resultant devices as nanosensors. These biosensors have been used for electrochemical experimentation and belong therefore in electrochemical biosensors. An exception is the development of stabilized polymerized lipid films on a filter paper that switch on and off their fluorescence and therefore belong to optical biosensors. Below we provide an overview of the most common techniques for the preparation of mini- or nano-biosensors based on lipid membranes. 

### 3.1. Metal Supported Lipid Membranes

**Figure 1:**
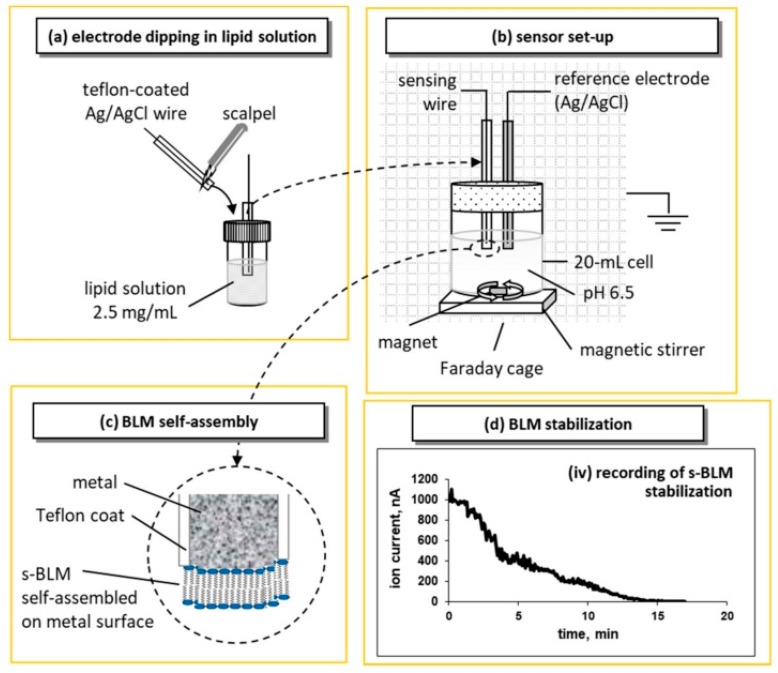
Representation of the device setup, and the lipid self-assembly process for the preparation of metal-supported stabilized bilayer lipid membranes (sBLMs; not drawn to scale) based on the original idea of [2]: (**a**) the tip of the sensing electrode is cut with a scalpel and immediately dipped in lipid solution before transferred in the electrolyte solution. (**b**) The electrochemical setup uses a two-electrode configuration (i.e., the sensing electrode and a Ag/AgCl reference electrode) in a magnetically stirred 20 mL cell. The set-up is placed in a grounded Faraday cage; 25 mV external DC potential is applied between the electrodes; the ionic current through the BLM is measured with a digital electrometer. (**c**) Upon immersion, the lipid drop attached to the tip of the wire is self-assembled into a bilayer; one layer is adsorbed on the metal surface and the other faces the electrolyte. (**d**) Recording of the ion current decrease during the self-assembly process. The recording starts with the immersion of the sensing electrode in the electrolyte solution (reprinted from Reference [13]).

Tien and Salamon [12] proposed a simple and reliable technique for the preparation of stabilized bilayer lipid membrane (sBLM) using the freshly cut tip of a Teflon coated metallic wire and taking advantage of the interaction between the amphiphatic lipid molecule with the nascent metallic surface. The procedure required the cutting of a Teflon-coated stainless steel metal wire (0.1–0.5 mm in diameter) while it was immersed in lipid solution in chloroform using a miniature guillotine. The tip of the wire is coated with lipid solution that turns into a lipid film; when transferred in electrolyte (0.1 M KCl), the lipid film spontaneously thins into a self-assembled lipid bilayer membrane (sBLM). A more recent and easier version of this approach is shown in Figure 1.

sBLMs have been fully characterized [2,14,15]. Device stabilization depends upon the diameter of the wires and the organic solvent used [14,15]. Wires of 0.25 mm diameter should be avoided due to increased sensor noise; the use of decane as a solvent should be also avoided as it enhances the tendency for “black” lipid membranes that do not provide reproducible results. Hexane solvent and silver wires with diameters of 0.5 and 1.0 mm provide BLMs that are mechanically and electrically stable for over 48 h. 

Some attempts have been made to model the potential profile across sBLMs and the structure of the lipid layer that faces the metal surface. A plausible theory involves the interactions of oxygen atoms of the phosphate groups of the lipid headgroups with the silver ions in the metal lattice [16,17]. Transmembrane ion mobility can be attributed to the presence of chloride ions at the space between the metal and the inner lipid layer. There could be two sources for chloride ions: through the lipid film during the initial BLM stabilization process and through the partial wire insulation [14,15]. Chloride would react with the silver metal to form silver chloride [14,15]. Potentiometric experiments (against a Ag/AgCl reference electrode) showed only small voltages (relative to a silver wire against a Ag/AgCl reference electrode) when the BLM had been removed using an organic solvent rinse. These results suggest that (a) the metal surface is possibly coated with a thin layer of silver chloride and (b) the lipid membrane actually consists of a network of nm-sized BLMs [18].

### 3.2. Stabilized Lipid Films Formed on a Glass Fiber Filter

The construction of stabilized in air lipid films that they were supported on Whatman glass fiber filters was previously given in detail in the literature [8]. These stable in air lipid membranes have given the opportunity to apply these devices for practical applications in real samples, such as the detection of aflatoxin M_1_ dairy products [9], etc. The lipid film was constructed on Whatman glass microfiber disks (Whatman GF/F, which had a 0.9 cm diameter and 0.7 µm nominal pore size) [8,9].

**Figure 2:**
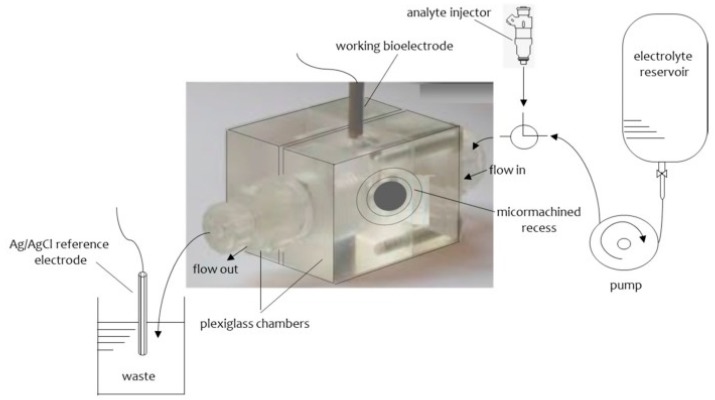
The experimental set-up used for the construction of stable in air lipid membranes supported on glass fiber filters (from Reference [13]).

The procedure of the construction of these glass fibers supported lipid membranes has been given in detail in the literature [8,9] and in brief is as follows: The experimental set up consisted of two Plexiglas chambers which were separated by a plastic Saran-Wrap. A 0.32 mm hole was made through this partition with a perforation tool. A microporous glass GF/F disk was positioned between the two plastic layers with the 0.32 mm hole in the center. The plastic partition was then clamped between two Plexiglas chambers. One of the chambers having a circular shape was connected with a plastic tube for the flow of the carrier solution. The second chamber was cylindrical and the upper hole had a circular shape with surface area of about 0.2 cm^2^ and its lower had an elliptical shape (diameters 0.5 and 1.4 cm parallel and vertical to the flow of the carrier electrolyte solution, respectively). The lower hole was placed at the center of the cylindrical cell. An external voltage of 25 mV d.c. was applied between two reference Ag/AgCl electrodes. A Keithley electrometer was used to measure the current. The samples were injected using a Hamilton repeating dispenser. A diagram of the apparatus is given in Figure 2. A drop of the lipid solution about. 10 µL was placed to the electrolyte surface (i.e., cylindrical cell). The level of the electrolyte solution was moved below the mm hole and then was raised. The current magnitude was at the time of the formation of the lipid bilayer on the order of pA and its bilayer structure was verified by the use of gramicidin D. 

### 3.3. Polymer-Supported Bilayer Lipid Membranes

The preparation of polymer stabilized has been recently described in literature [10,11]. Initially the polymerization was made by heating the lipid mixture at 60 °C; however, later on UV irradiation was used. The reason is that the former technique deactivates the enzymes, antibodies, or natural receptors. Physicochemical methods, such as DSC, IR, or Raman spectrophotometry, indicated that the polymerization process requires about 4 h. The lipid membranes which were prepared using this technique were stable outside the electrolyte solution for about one month.

**Figure 3:**
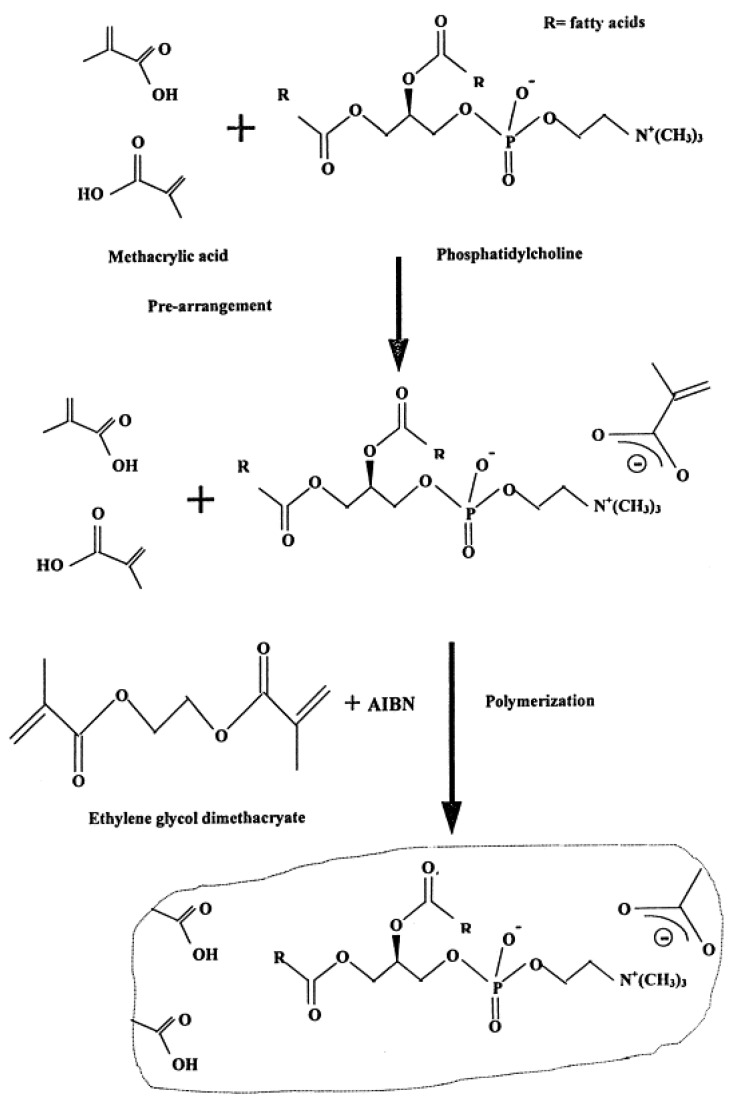
A schematic version of polymerization stage and preparation of polymerized lipid membranes (from Reference [19] with permission).

The construction of these stabilized lipid films has been described in detail in the literature and in brief is as follows [10,11]: 0.8 mL of a mixture which contained 4% *w*/*v* egg PC in n-hexane was mixed with 0.07 mL of methacrylic acid, 0.8 mL of ethylene glycol dimethacrylate, 8 mg of 2,2’-azobis-(2-methylpropionitrile) and 1.0 mL of acetonitrile. A volume of 0.15 mL of this suspension was placed on a Whatman glass GF/F microfilter disk (diameter about 0.9 cm and had nominal pore size of 0.7 µm); the filter was then irradiated with a UV deuterium lamp for about 4 h. A simplified diagram of the set up used is presented in Figure 2. Figure 3 shows a schematic version of polymerization stage and preparation of polymerized lipid membranes.

### 3.4. Polymer Lipid Films Supported on Graphene Microelectrodes

Graphene nanomaterials have been extensively used for the construction of nanosensors due to their unique physicochemical properties which are good sensitivity, excellent mechanical and electrical properties, enhanced thermal stability, large surface-to-volume ratio, improved biocompatibility, high electron-transfer rates, limited toxicity, and bio-safety. Their large surface-area-to-volume ratio provides device size reduction and faster response times; the former might be proven critical for commercialization whereas the latter allows for lower detectabilities while adequately handling biofouling problems. Several nanobiosensors have been described using enzymes and antibodies. A reliable system presented involves stabilized lipid films wrapped around a copper wire containing graphene nanosheets [20,21]. These nanosensors have been implemented in the rapid detection of food toxicants, environmental pollutants and toxins in real samples, such insecticides [21], naphthalene acetic acid [22], cholera toxin [23], and saxitoxin [24].

The construction of graphene microelectrodes has been described in the literature and in brief is as follows [20,21,22,23,24]: a homogeneous graphene dispersion (∼0.4 mg/mL) is prepared using *N*-methyl-pyrrolidone (NMP) and mild sonication for 180 h followed by centrifugation at 700 rpm for 2 h. The graphene dispersion was placed onto a copper wire (0.25 mm in diameter) and the organic solvent was evaporated. The extended sonication has results in a good fraction of monolayer sheets but with smaller lateral sizes.

**Figure 4:**
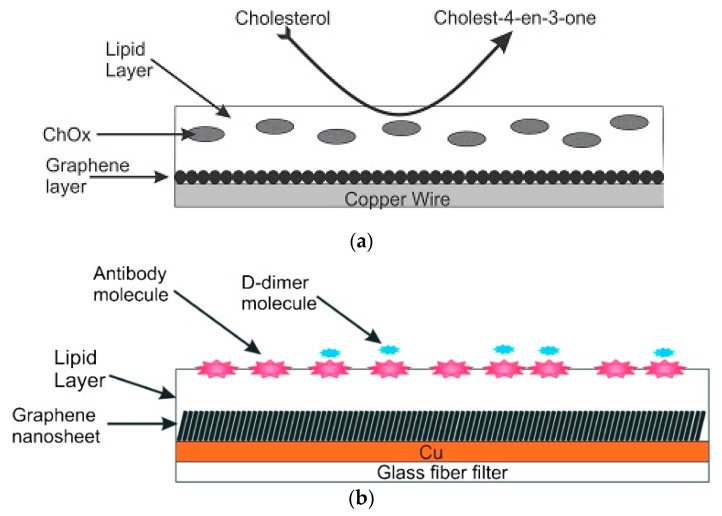
(a) Set-up of potentiometric experiments for the determination of cholesterol (from Reference [25] with permission). (b) Set-up of potentiometric experiments for the determination of D-dimer (from Reference [26] with permission).

**Figure 5:**
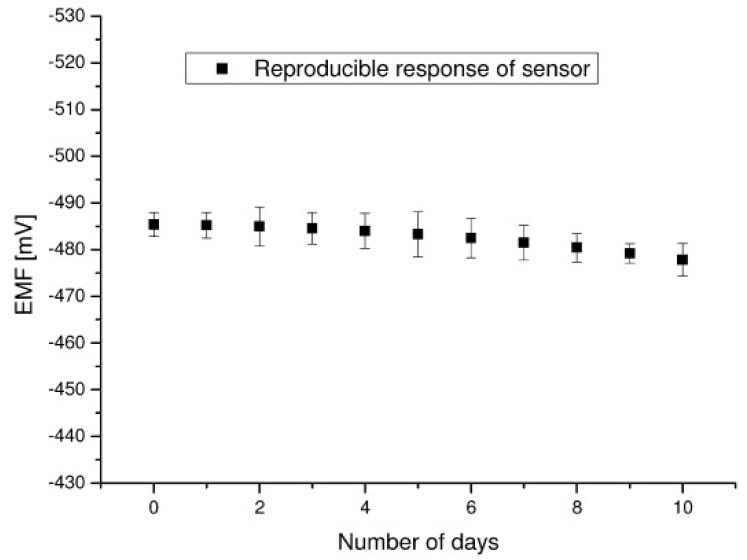
Carbofuran sensor reproducibility/reusability at room temperature after 2–3 h span in 100 mM carbofuran solution (from Reference [21] with permission).

Stabilized lipid films were constructed as described in [10,11] which was previously described. The enzyme, antibody or receptor (“receptor”) was incorporated in these lipid membranes prior to polymerization by spreading 15 µL of the “receptor” suspension over the polymerization mixture. The polymerized lipid membrane was mounted onto the copper wire containing graphene nanosheets to produce the nanosensor. Figure 4 and Figure 5 show a diagram of the experimental set-up used in these previous works.

### 3.5. Fabrication of Biosensors with Nanoporous Lipid Membranes

The nanoporous lipid membranes have reported about a decade ago. The construction of the nanoporous lipid membrane is made as follows [27]: the biosensor is constituted of a free-standing silicon nitride (Si_3_N_4_) supporting membrane. The membrane has nanoapertures on which the lipid film will be incorporated; the lipid film will host the proteineous receptors. These proteineous receptors are altered so that they can react with the lipid film and an ion channel opens by binding to a ligand. The “ion channel” results to an ion current (on the order of pA) which is measured with Ag/AgCl electrodes.

Nanoporous alumina or aluminum anodized oxide membranes have been reported to be utilized in a wide range of applications [28,29,30,31,32,33,34,35,36]. In some instances the nanoporous membrane was in the shape of a film [31], or sputtered onto the surface and then anodized [33]. Alumina membranes are also commercially available. Alumina membranes have a large range of excellent properties which are the following: non-conductivity, well defined nanopores, small pore size, high pore density, and ease of functionalization [32].

Gold [36,37], silver [34], titanium oxide [38], and glass [39,40,41,42,43] inorganic membranes were also reported that were used for nanoporous membrane construction. Glass fiber membranes were reported to transport fluids by capillary action [40,41,42,43]. Silicon nitride was reported in hybrid membrane structures to act as a support for organic membranes [44]. Al_2_O_3_, Ag, and gold-coated polycarbonate track-etched (PCTE) membranes were repeatedly used for surface enhanced Raman spectroscopy [34]. 

## 4. Applications of Lipid Film Based Biosensors in Food Analysis and Environmental Monitoring

An atrazine lipid film biosensor was reported in the literature with mM detection limits [45]. When atrazine interacted with bilayer lipid membranes (BLMs) free of solvent, a transient ion current signal appeared (i.e., transient) and lasted seconds; this transient reproducibly appeared within 1 min after the explosion of the films to atrazine. The sensitivity of the biosensor increased by using 35% (*w*./*w*.) DPPA in the lipid mixture and Ca^2+^ in the electrolyte solution; calcium ions promoted changes in the phase structure of the acidic lipid (i.e., DPPA) and therefore increased the magnitude of the signals. Similar results were obtained by using platelet-activating factor (PAF; an ether analog of PC) in membranes. 

The flow injection analysis of mixtures of the triazine herbicides (i.e., simazine, atrazine, and propazine) on phosphatidyl choline/dipalmyitoyl phosphatidic acid filter-stabilized BLMs has been reported in the literature [46]. An ion current transient appeared after exposure of membranes to a mixture of these herbicides in less than two min after injection. The peak heights was linearly correlated to the herbicides concentration, which were detected at µM range. Repetitive cycles of injections exhibited that the signal did not decrease. The appearance time of these signals varied for each herbicide and was larger on the order of simazine, atrazine, and propazine; this has allowed the simultaneous determination and analysis of mixtures of these herbicides.

Lipid membranes devices were utilized for the determination of carbofuran using flow injection analysis (FIA) techniques [47]. The principle was based on the inhibition degree and reactivation of enzyme when the substrate was injected. Carbofuran could be detected in the range from 10^−7^ to 10^−9^ M. Proteins and lipids were examined as interferents and no such an interference was noticed. The sensor has been used in a wide range of food samples, such as fruits, vegetables, and dairy products. A recovery of 96% to 106% was noticed which shows that there were no interferences from the matrix effects.

An electrochemical carbofuran device on graphene nanosheets in which stabilized lipid films were immobilized has been reported in the literature [21]. This electrode was utilized to construct a carbofuran biosensor that used an artificial receptor (i.e., resorcin[4]arene). The limits of detection was down to nM and times of response were about. 20 s. This device was easily constructed and shown an excellent reproducibility and reusability as it is shown in Figure 5. The selectivity and other electrode characteristics such as long shelf life and sensitivity were also good. The electrode had a slope of about 59 mV/decade in the carbofuran concentration between 10^−6^ and 10^−3^ M.

A synthetic “receptor” attached on stabilized lipid membranes using glass fiber disks was reported in the literature [48]. The stabilized lipid membranes were modified with calixarenes. The method had a high sensitivity and selectivity so that it can be applied for the fast detection of insecticides in fruits and vegetables [48]. Other biosensors that were reported include a chemical sensor for the selective and rapid determination of hormones in foods which included naphthalene acetic acid [49] and also a device for the rapid determination of zinc in waters [50].

**Figure 6:**
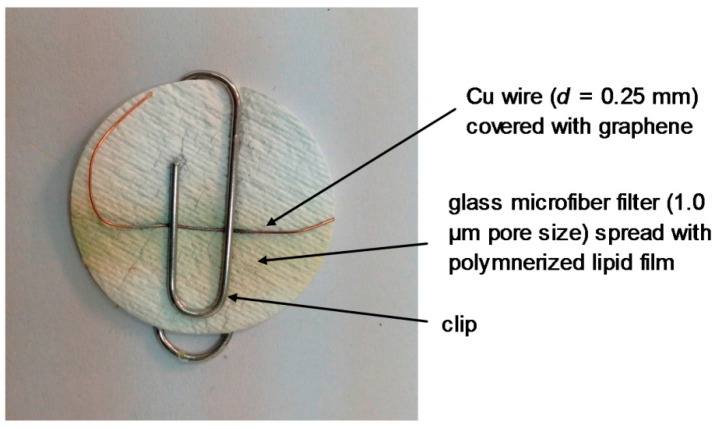
Photograph of the lipid film sensor incorporated on graphene for the potentiometric detection of urea (reprinted from Reference [20]).

A potentiometric urea lipid membrane minisensor incorporated on graphene nanosheets appeared in the literature [20]. The structural characteristics of graphene nanosheets were explored by using atomic force microscopy (AFM) and transmission electron microscopy (TEM). The pre- and post-conjugated surfaces of graphene were investigated UV-Vis and Fourier transform IR (FTIR). A potentiometric urea device was constructed (Figure 3) that has shown excellent reproducibility and reusability, good selectivity and rapid times of response (ca. 4 s), long shelf life, and a good sensitivity with a slope 70 mV/decade on concentration range of urea from 1 × 10^−6^ M to 1 × 10^−3^ M. Figure 6 shows a photograph of the lipid film sensor incorporated on graphene for the potentiometric detection of urea. 

A potentiometric urea biosensor appeared in the literature by immobilizing urease on eggshell [51]. Eggshell was treated with polyethyleneimine (PEI) to gain polycation properties. Urease was then immobilized on the PEI treated eggshell membrane. SEM studies were performed to explore the alterations of the surface structure and an FTIR study was performed to find the alterations of the IR spectra. The device has shown a sigmoidal response for the concentrations of urea between 0.5 and 10 mM with a response time of 120 s. This biosensor was stable for 2 months when stored in buffer even at room temperature.

A biosensor for naphthalene acetic acid (NAA) was reported in the literature and was based on stable lipid membranes on a methacrylate polymer using glass fiber disks with incorporated auxin-binding protein 1 receptor [22]; the sensor was evaluated using real samples of fruits and vegetables. NAA was injected into the flowing carrier electrolyte solution and the flow stopped until an ion current transient was obtained; the height of the ion transient was related to the hormone concentration with mM detection limits. The time of analysis was ca. 5 min. Interferences studies were performed with wide range of interferents. This study has shown that there were no interferences at levels that existed in real food samples. The sensor was evaluated for the detection of NAA in fruits and vegetables and excellent reproducibility. 

Electrochemical aptasensors based on glassy carbon electrodes in which electropolymerized Neutral red and polycarboxylated macrocyclic ligands were reported recently in the literature [45]. DNA aptamers were immobilized and aflatoxin B1 (AFB1) was detected with high sensitivity and selectivity [52]. The cathodic peak current of the probe was decreased (using Cyclic Voltammetry, CV) and electron transfer resistance determined measured by by Electron Image Stabilization (EIS) was increased. The detection limit was 0.1 nM for CV and 0.05 nM for EIS methods, respectively. AFB1 was detected in peanuts, cashew nuts, white wine, and soy sauce and the recovery was 85–100%.

A strategy was described in the literature that was based on monitoring the alterations of ion current through a lipid membrane with immobilized DNA probes caused by interaction of these membranes with hydrazine compounds [53]. A sBLM that was composed of phosphatidylcholine was formed on a silver metal electrode. The s-DNA that were used were thymidylic acid icosanucleotide and were terminated with a C-16 alkyl chain so that to help the incorporation into sBLMs (dT_20_-C_16_) and deoxyadenylic acid icosanucleotide (dA_20_). These sBLMs were found to interact with hydrazines, thus making feasible to monitor ppb levels of hydrazine, methylhydrazine, dimethylhydrazine and phenylhydrazine. This BLM/DNA sensor exhibited an excellent sensitive, selective, rapid performance. Through this route, a portable biosensor for monitoring these toxicants was constructed.

The scope of a paper that appeared in the literature is the development of nanofluidic biosensor that is able of detecting single molecules [54]. The detection principle was based on the measurement of the ion current through an ion channel when a molecule transports through the channel; the channel has a diameter on the molecule size. The walls of these channels are coated with a lipid bilayer, which acts as two-dimensional liquid and therefore the transport properties of such channels are improved. Presently, this property of lipid membranes was used to developing a technique to detect single-stranded DNA (ssDNA) through these channels that had a luminal radii of 5–7 nm. It was shown that using low ionic strength, when DNA molecule enters such channel, this is accompanied by an increase of its ion conductivity; this effect can be controlled by the polarity of the applied voltage. The peak height of the current increase permits to evaluate the number of DNA molecules inside the channels. It was also shown that when the DNA molecules were adsorbed on the lipid bilayer surface, the membrane cylinder behaves as a voltage-sensitive ion channel. 

A work that reports a BLM-based nucleic acid biosensor supported by modified patch-clamp pipette electrode was developed for staphylococcus enterotoxins B (SEB) gene [55]. Hydrophobic dodecane tail (C_12_) modified 18 bp single-stranded DNA (ssDNA) probe was immobilized on the membrane to yield linear correlations. The sensor was constructed by selecting the ssDNA probe as the signal sensing element with the concentration of 273.65 ng/mL. The electrochemical performance of the biosensor for SEB detection was studied, showing a linear relationship between the current and ln(concentration) from 20 to 5000 ng/mL, with a detection limit of 20 ng/mL. In addition, the biosensor has shown a specific response to SEB gene and no significant current alteration in the absence of the SEB gene. AFM images were used to evaluate the microstructure of BLMs, ssDNA immobilized on BLMs and BLMs after hybridization. The sensor could be developed in a reliable tool for the detection of *Staphylococcus aureus*, which produce SEB.

A paper appeared in the literature that describes the electrochemical interactions of cholera toxin with polymerized lipid films in which ganglioside GM1 was immobilized [56]. The analyte was injected into the flowing streams of a carrier electrolyte solution, the flow of the solution stopped for 5 min and an ion current transient was obtained. The peak height of the ion current could be related to the concentration of cholera toxin with detection limits of 0.06 µM. 

**Figure 7:**
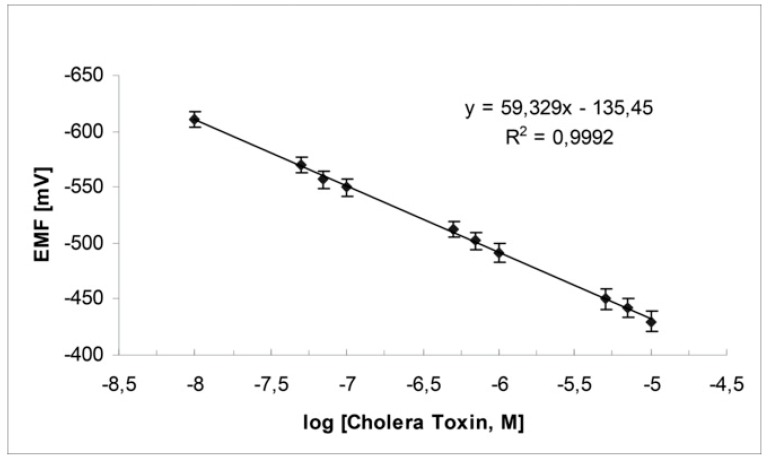
Calibration graph for cholera toxin determination using the potentiometric cholera toxin device (from Reference [23] with permission).

Switching to stabilized polymeric lipid films on graphene nanosheets, ganglioside GM1 has given better results, i.e., response time of ca. 5 min, and detection limits of 1 nM [23]. The proposed device is constructed easily and shows excellent reproducibility, reusability, selectivity, long shelf life, and sensitivity. The slope of the electrode is 60 mV/decade of toxin concentration as shown in Figure 7. The method was evaluated, implemented, and validated in lake water samples. 

A chemiluminescence biosensor formed on a supported lipid layer with immobilized ganglioside GM1 was provided in the literature reported for cholera toxin [57]. The stabilized lipid film was constructed as a biosensing interface via spontaneous spread of ganglioside-incorporated phospholipid vesicles on the octadecanethiol-coated gold surface [57]. The specific interaction of multivalent toxin by ganglioside GM1 molecules has given the opportunity for implementing the sensor in a sandwiched format using a GM1 and horseradish peroxidase (HRP) functionalized liposome probe, where the presence of the toxin could be detected via the HRP-catalyzed enhanced chemiluminescence reaction. The advantages of this technique over conventional strategies, are plenty such as easiness of construction and renewal of the sensing surface, low background noise, and effective immobilization of recognition species. Cholera toxin could be detected using this device within the concentration range between 1 pg mL^−1^ and 1 ng mL^−1^ with a detection limit of 0.8 pg mL^−1^.

**Figure 8:**
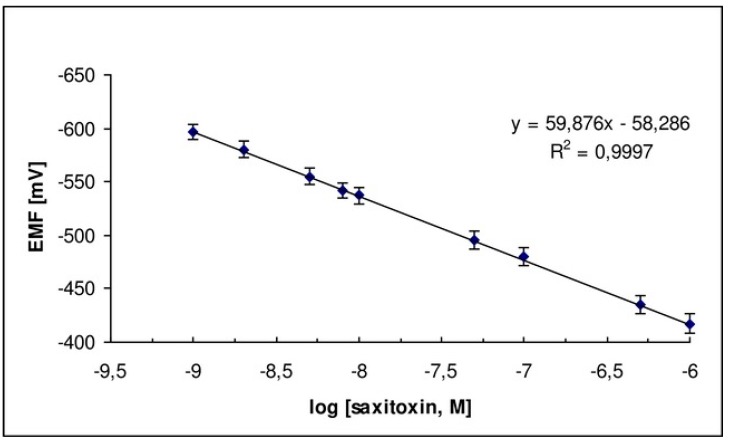
Calibration graph for STX determination using the saxitoxin potentiometric biosensor [from ref. [24] with permission].

A potentiometric saxitoxin device which was based on graphene with a lipid membrane and in which anti-STX (which is the natural saxitoxin receptor) was immobilized was recently reported in the literature [24]. An excellent selectivity and sensitivity for the determination of saxitoxin, rapid times of response (about 5–20 min), and detection limits of 1 nM were noticed. The construction of this biosensor was easy; the sensor exhibited good reproducibility, reusability and adequate storage stability with a slope of about 60 mV/decade, as shown in Figure 8. The method was evaluated and validated in lake water and shellfish samples. 

A novel electrochemical biosensor based on a supported polymeric lipid membrane with immobilized Sheep anti-PCB antibody for the rapid determination of arochlor 1242 in flowing solution streams (FIA systems) has been reported in the literature [58]. The antigen was injected into the flowing streams of a carrier electrolyte solution. The experiments were performed in a stopped-flow method; the lipid mixtures were constituted by 15 % (*w*/*w*) DPPA and 85% of DPPC to provide one single transient current signal with a peak height related to the concentration of arochlor. Repetitive cycles of injections of antigen were made to find out the maximum number of cycles and the results have shown that this number of injections were 5.

A sensor that is appropriate for the rapid detection of sucralose was described in the literature [59]. The device was based on sBLMs that were constructed from phosphatidylcholine. An ion current increase was the result of sBLMs interactions with sucralose. Differential scanning calorimetry (DSC) was utilized to explore the mechanism of signal generation. The signal generation was due to changes of the electrostatic fields of the lipid film. The ion current signal were related to sucralose concentration with µM detection limits. The detection range was between 5–50 µM and the response times on the order of a few seconds. This biosensor was used for the rapid detection of this sweetener in granulated sugar substitute products.

Flow injection analysis was used for the determination of mixtures of the artificial sweeteners acesulfame-K, cyclamate, and saccharin using filter-supported BLMs [60]. A transient current with duration of seconds appeared in less than 1 min after exposure of the lipid membranes to the artificial sweeteners. The peak height of this signal could be linearly correlated to the concentration of artificial sweeteners, at the µM concentration range. The appearance time of these signals varied for each artificial sweetener and increased in the order of cyclamic acid, acesulfame-K, and saccharin. This fact has permitted the simultaneous determination of these sweeteners in mixtures. Interference studies were performed and these studies have shown no interference from a wide range of compounds found in foods. The method was evaluated in real food samples (i.e., artificial sweetener tablets, diet soft drinks, wines, and yogurts) that contain mixtures of artificial sweeteners. The results were compared with results of analysis using an Official Method of Analysis and no differences were found between the two methods.

A BLM glucose device which was based on glucose oxidase deposited on platinum that has been modified with several polymers was reported in the literature [61]. Excellent results were received for a mediated system in which the BLM was constructed on a Pt support which was covered with a layer of evaporated Nafion with incorporated ferrocene. The stable, selective, and sensitive response appears to be very promising for practical applications.

A nanostructured electrochemical biosensor was developed for screening estrogenic substances using only the estrogen receptor (ER) and reported in the literature [62]. ERs were deposited on sBLM modified with Au nanoparticles, and the electrochemical responses of the modified electrodes were investigated by CV and EIS. The results showed that this device could detect 17β-estradiol at the concentration range between 5 and 150 ng/L and had a DL of 1 ng/L. It was also found that Au nanoparticles could enhance the sensitivity and stability of the sensor. The sensor was used for the screening the estrogenic activity of waters and the results have shown to have good agreement with those obtained by MCF-7 cell proliferation assay.

## 5. Conclusions and Future Prospects

The present paper describes a variety of approaches and strategies to construct nanosensors based on lipid film technology and implement them for food and environmental analyses. The recent technological advances include the engineering of stabilized supported lipid film on graphene nanoelectrodes with an incorporated “receptor” of any kind, natural or artificial. These films remain stable in air and are suitable for the development of portable devices for in the field applications. The sensors exhibit detection limits in the nM concentration range. In effect, a portable unit that can be used for in-field and market applications might be developed in the near future.

The results have shown that a variety of lipid film based detectors can be reused after storage in air, even after few months, and can be reproducibly fabricated with simplicity and low cost. These nanosensors have fast response times and are easy to construct at quite lesser cost than chromatography-based instrumentation; they can be also used as rapid hand-held detectors complimentary to these methods for in-field and market measurements in foods and for environmental monitoring.

The present review describes biosensors based on lipid film technology that can be used for the rapid detection of food toxicants and environmental pollutants such as toxins, carbamates, hormones, polycyclic aromatic hydrocarbons, etc., and highlights their advantages which are high sensitivity and selectivity, rapid response times, portability, etc. It is of common sense that the use of nanotechnology to construct lipid membrane based biosensors will provide devices with even improved characteristics. 

The lipid membrane technology has made significant technological advantages as compared to the traditional based detection methods (i.e., chromatography or spectroscopy). Still, despite this fact, there are numerous issues that research should be targeted and these include portability so that the devices are hand-held, the analysis of a large number of samples simultaneously, and finally these devices have to be used by non-skilled personnel. Also portable and handheld biosensors, for example, such as dynamic DNA and protein arrays for rapid and accurate detection of pathogens, are a few typical examples on how this sensor technology can provide advances in science and the use of vesicles will advance this field of science one step ahead.
